# Digital Eye Strain and Its Risk Factors Among a University Student Population in Jordan: A Cross-Sectional Study

**DOI:** 10.7759/cureus.13575

**Published:** 2021-02-26

**Authors:** Yazan Gammoh

**Affiliations:** 1 Faculty of Allied Medical Sciences, Department of Optometry Science, Al-Ahliyya Amman University, Amman, JOR

**Keywords:** computer vision syndrome, digital devices, young adults

## Abstract

Background: Several ocular and visual symptoms resulting from use of digital devices are collectively known as digital eye strain (DES) or computer vision syndrome (CVS). Few studies exist on the prevalence of DES among young adults in the Eastern Mediterranean region.

Objectives: This study aimed to investigate the prevalence and severity of digital eye strain among a university student population in Jordan.

Methods: A cross-sectional study was conducted with students enrolled at Al-Ahliyya Amman University in Jordan. DES was evaluated using the Computer Vision Syndrome Questionnaire (CVS-Q), which was filled out by students who were approached at random and signed an informed consent to participate in the study. Information related to the type, intensity of usage, settings of digital device (DD) used, and post-device use student-reported physical complaints were recorded.

Results: Data from 382 students were analyzed. Prevalence of CVS was 94.5%, with tearing being the most prevalent symptom (59%), while double vision was least reported by students (18.3%). DD use for more than six hours per day was reported by 55.5% of the sample size, and 30.7% of the students reported pain in joints of fingers and wrists after using a mobile phone.

Conclusion: CVS is highly prevalent among university students in Jordan. With the increased dependence on online education due to coronavirus disease, safe habits in digital device use are recommended.

## Introduction

In this day and age, computers and visual display terminals have become an integral part of our daily lives. Collectively referred to as digital devices (DD), smartphones, tablets, electronic book readers, and computers have significantly increased in recent years and resulted in several ocular and visual symptoms related to their use, conjointly now known as digital eye strain (DES) or computer vision syndrome (CVS) [1]. Common symptoms of the aforementioned include eye strain, headache, blurred vision as well as neck or shoulder pain that often increases in severity with the amount of video display terminal (VDT) use [1]. The increased use of digital screens not only increases the odds of developing CVS, but also occupational overuse syndrome (OOS), an injury to fingers and wrists caused by repetitive movements, headache as well as psychosocial stress [2]. In fact, it is estimated that around 60 million people suffer from CVS globally and that a million new cases of CVS occur annually [3].

It has been shown that 30% of workers use computers all the time during their working days based on the findings of the European Working Conditions Survey (EWCS, 2010) [4], and 25% of computers between one-fourth and three-fourths of the time spent during the working hours [3]. Moreover, computer use is not restricted to adults, as a recent study involving over 2000 American children between the age of eight and 18 years found that, in an average day, children spend about 7.5 hours using entertainment media, 4.5 hours watching TV, 1.5 hours on a computer and over an hour playing video games [5].

Students of any age have gradually transitioned to computer-based learning believing that it is a more attractive option compared to conventional classroom teaching, not to mention that most schools and universities nowadays have smart boards and require online submission of homework/assignments. This paradigm shift has penetrated among youth [6]. The prevalence of deteriorating effects of prolonged DD use among university students translates to CVS/DES symptoms, especially when the visual demands of a given task exceeds the visual abilities of a student to comfortably perform the task at hand [7].

Studies have shown that eye-related symptoms are prominent despite little guidance on the operational definition of CVS or DES in literature. This is evident by the broad difference in regards to the criteria used to determine when an individual is considered to have developed symptoms of DES, owing to the lack of validated measurement instruments [8].

Despite the increasing worldwide attention towards the escalating prevalence of CVS, there are few studies in the literature examining the prevalence of CVS among young adults in the Eastern Mediterranean region [2,4,9]. In addition, most of the studies relied on only one major of students (e.g., medicine students) which could not represent the diverse university population [2,4,9]. Moreover, there are no published studies concerning CVS in Jordan. The urge to perform such studies in Jordan is attributed to the fact that residences of low-income countries are more prone to eye-health complications such as blindness, uncorrected refractive error and glaucoma. As such, we believe that CVS prevalence patterns would be different, not to mention that the development of CVS and DD use is related to the lifestyle and cultural variations which are immensely different among different regions also.

This study aimed to investigate the prevalence and severity of digital eye strain among a university student population in Jordan. The study is meant to provide evidence to the eye-care professionals, educators and policymakers to design and implement an intervention strategy to alleviate the symptoms associated with DD usage.

## Materials and methods

An observational, cross-sectional study was conducted between February and March 2020 with students enrolled at Al-Ahliyya Amman University in Jordan. At the beginning of the study, the university students’ population was 7000 as per information obtained from the Registrar office. A sample size of 383 was deemed to be representative of the university student population using GRANMO version 7.12 [10], and assuming a 50% predictive prevalence [11], a 95% confidence interval level, a ±5% margin of error and a 5% replacement rate.

Students were approached at random on campus, where the investigators invited the students to participate in the study after verbally explaining the study aspects in addition to providing them with written details of the study. The participants filled out the questionnaire at the university laboratory and the investigators provided assistance if needed. In cases where multiple answers were applicable, such as type of DD used, participants were asked to specify the major answer, for instance the most frequently used device (based on hours use per day) to avoid repetition. Four hundred and fifteen students agreed to participate, however, 382 surveys were included in the data analysis after excluding the participants who met the exclusion criteria. Each participant underwent a routine eye examination and a review of medical and ocular history performed by licensed practitioner (Investigator YG).

All university students were eligible to participate in the study if they had an un-corrected or a best-corrected (with spectacles or contact lenses) binocular distance visual acuity of 0.0 logMAR (Snellen equivalent of 6/6) or better measured at 4 metres using the Early Treatment of Diabetic Retinopathy Study (ETDRS) Chart (Precision Vision, La Salle, IL, USA). Exclusion criteria included a history of ophthalmic surgery, active ocular disease, or lack of clear ocular media. In addition, participants who did not understand the instructions or did not sign a consent form were excluded from the analysis.

Participants’ demographics such as age and gender were recorded in the study form. Computer vision syndrome among the participants was evaluated using the Computer Vision Syndrome Questionnaire (CVS-Q) [7]. The CVS-Q is a self-administered questionnaire that assesses the frequency and intensity of sixteen symptoms associated with use of digital screens. A questionnaire that was administered on a population similar to the sample in the current study was also used [2]. Moreover, post-device use self-reported physical and ocular complaints were also obtained from the participants. 

Data were entered into Microsoft Excel spreadsheets and then analyzed using the Statistical Package for Social Sciences (SPSS) software version 25 (IBM Corporation, Armonk, NY, USA). Participants were deemed to suffer from CVS if they score a severity of ≥6 using the CVS-Q [7]. Numbers and percentages were calculated to summarize categorical and nominal data. The chi-square test was used for categorical outcomes. P-values less than 0.05 were considered statistically significant.

Ethical approval (approval number: AAU-2/2020) was obtained from the Ethical Committee of Faculty of Allied Medical Sciences at Al-Ahliyya Amman University. The study was conducted as per the tenets of the Declaration of Helsinki of the year 1964 and its later amendments and all participants signed a consent form. Participants were free to withdraw from the study at any point.

## Results

A total of 415 students participated in the study, however only data from 382 participants (92% of the respondents) were included while 33 students were excluded (8% of the sample). Nevertheless, the study sample population can be considered to be representative of the university population as stated earlier in the methods section. The mean age (±1 standard deviation) of the participants was 21.5 years (±1.834), ranging from 18 to 24 years, with 39.1% (n=149) of the sample population being males and 60.9% (n=233) females. The male:female ratio was 1:1.56.

**Figure 1:**
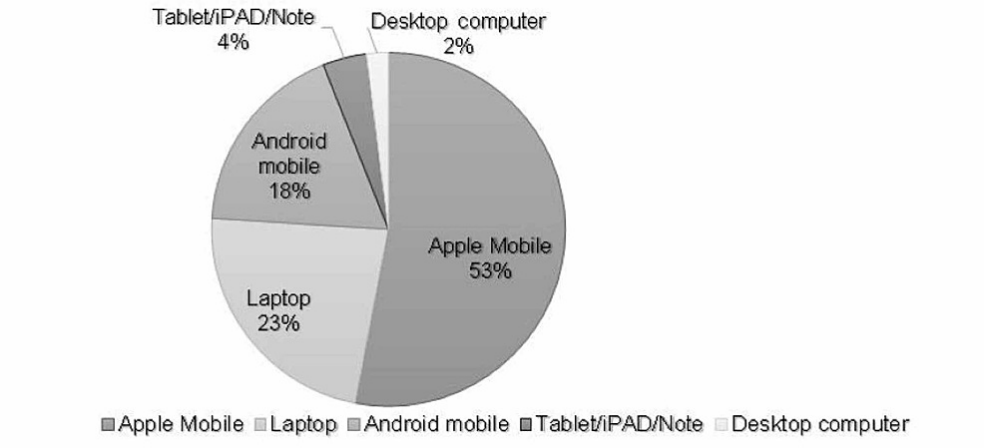
Types of digital devices used by university students (n=382).

Figure 1 shows the types of digital devices (DD) used by the participants. Apple® mobile phones (smartphones); regardless of the generation, were the most commonly used device with 52.9% of the sample (n=202) reported using them. However, ordinary computer screens (desktop screens) were only used by 2.1% of the students (n=8).

The habits and routine of DD use by students are shown in Table 1. It was observed that the majority of the participants (63.5%, n=242) were using digital devices for more than four years. Interestingly, only 10.7% of the students (n=41) reported using DDs for less than one year. Regarding the number of hours spent using DD per day, more than half of the students (55.5%, n=212) reported spending more than six hours. On the other hand, only five students (1.3%) spent less than one hour per day. 

**Table 1:** Digital devices usage habits among university students (n=382). DD= digital device. %= percentage

Usage Habit	Duration	Frequency (%)
Years of current usage of DD	<1	10.7
	1-<2	13.8
	2-<3	22.9
	3-<4	15.6
	≥4	36.5
Hours spent per day using DD	<1	1.3
	1-<2	4.2
	2-<3	5.5
	3-<4	13.6
	4-<6	19.9
	≥6	55.5
Hours spent are	Continuous	36.1
	Intermittent	63.9
DD are mostly used during	Day	46.3
	Night	53.7

As shown in Table 1, more than half of the students (63.9%, n=244) reported taking breaks of 20 seconds every 20 minutes and looking at a distance of approximately 20 feet during the use of DD, while the rest (36.1%, n=138) conveyed using DD continuously i.e., without taking a break as defined above. Slightly more than half of the students (53.7%, n=205) reported using DD mostly during the night while the rest used DDs during the day.

**Figure 2:**
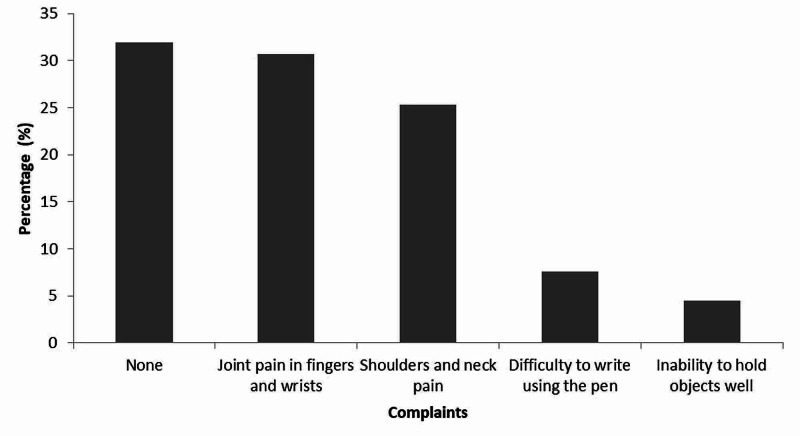
Upper body musculoskeletal complaints by university students after using smart phone for prolonged hours (percentage, n=382).

Complaints reported by students after using a smart phone for prolonged hours are shown in Figure 2. It was observed that 31.9% (n=121) of the participants in this study had no complaints, while 30.7% (n=117) had joint pain in fingers and wrists, followed by shoulders and neck pain (25.3%, n=96). The least reported complaint was inability to hold objects well (4.5%, n=17) followed by difficulty to write using a pen (7.6%, n=29).

**Figure 3:**
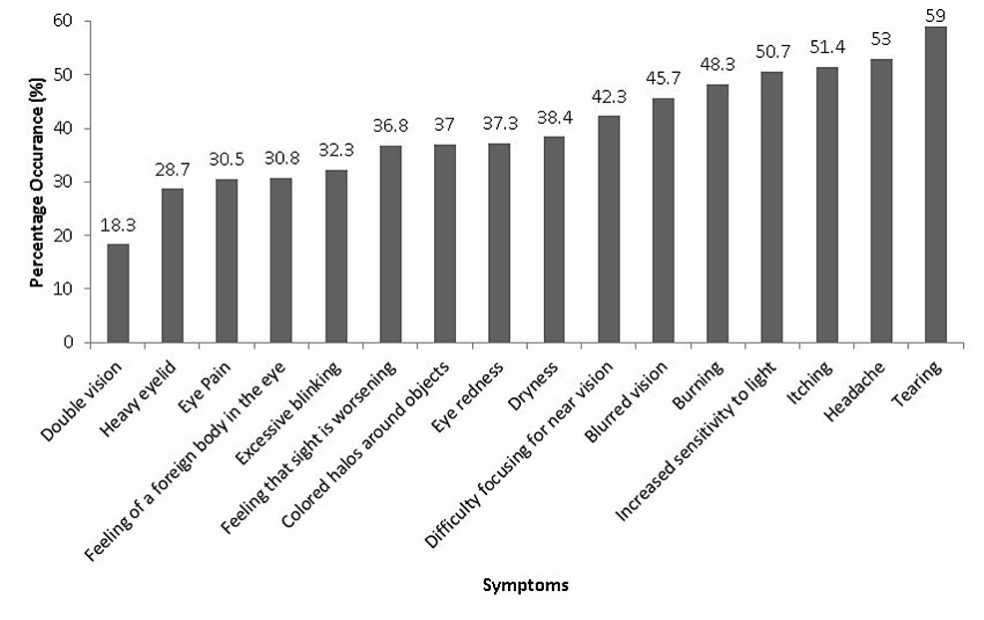
Occurrence of Computer Vision Syndrome symptoms in university students (n=382).

Most of the students (94.5%, n=361) were deemed to suffer from CVS as they had a severity score of ≥6 for symptoms reported using the CVS-Q. The prevalence of symptoms using a DD is shown in Figure 3. The symptoms most frequently reported by the students were tearing (59%, n=225), headache (53%, n=202) and increase sensitivity to light (51%, n=194). Students complained the least from heavy eyelids and double vision, with a prevalence rate of 29% (n=11) and 18.3% (n=70), respectively. The rest of the symptoms had a prevalence rate range between 30.5% and 48.3%.

Table 2 reveals that there is a relation between hours spent on DD and CVS symptoms, with 93.9% (n=199) of the students who used DD for more than six hours total during the day suffering from CVS. As it was not possible to ascertain the accuracy of self-reported number of hours per day of use of DD, the students were asked to share with the investigators the number of hours spent on DD as reported by the DD through its internal software, if applicable. It is worth mentioning that participants were asked to give the average number of hours spent on DD as inter-individual variations are expected.

**Table 2:** Association between hours spent on DD and CVS symptoms DD= digital devices. CVS= computer vision syndrome. SE= standard error.

	Do not have CVS				Have CVS*				
Number of hours	Count	SE	Row N%	SE	Count	SE	Row N%	SE	P value**
<1	0	.	0.0%	.	5	2	100%	.	
1-<2	2	1	12.5%	8.3%	14	4	87.5%	8.3%	<0.001
2-<3	3	2	14.3%	7.6%	18	4	85.7%	7.6%	<0.001
3-<4	7	3	13.5%	4.7%	45	6	86.5%	4.7%	<0.001
4-<6	9	3	11.8%	3.7%	67	7	88.2%	3.7%	<0.001
≥6	13	4	6.1%	1.6%	199	10	93.9%	1.6%	<0.001

Students were asked if digital screens can affect lifestyle and eye health, with 69.2% of the participants (n=265) agreeing with the question. In addition, students were asked if they are willing to decrease DD hours to prevent CVS, with 59.2% (n=226) of the sample responding that they would decrease DD use. There was a statistically significant difference (P<0.05) between genders regarding their willingness to decrease their DD usage hours, where 66% of females agreeing to change their habits compared to 47.7% of male students.

## Discussion

The current study used the CVS-Q, a Rasch-based questionnaire that investigates the frequency and intensity of 16 symptoms related to CVS [7]. Using the CVS-Q, a severity score of six or more indicates that the respondent suffers from CVS. Based on this score, 94.5% of university students surveyed in this study can be deemed to have CVS. Previous studies on prevalence of CVS among university students in the Eastern Mediterranean region also indicated a high prevalence, with 97.3% prevalence among medical and health sciences students in Saudi Arabia [12]. It is also estimated that 86% of medical students in Egypt complain of at least one symptom related to CVS [2]. However, none of the above studies used a validated questionnaire such as the CVS-Q. A prevalence rate of 72% was recorded among university students with different specialties in the United Arab Emirates (UAE) [13]. The prevalence reported in the UAE is much lower than the current study despite the similarity in the age range and the various specialties of students. The difference could be attributed to the origin of students in the UAE study, where more than half of the students surveyed were not of Middle Eastern origin. Reports on university students from other parts of the world show a generally high prevalence, despite being lower than most of the studies available from the Middle East. A prevalence rate of 71.6% was reported among medical students in Nepal [14], while 80.3% of students in India majoring in medicine and engineering suffer from CVS [3]. The prevalence of CVS in the current sample could be one of the highest recorded among this age group. However, it is not easy to compare reports of prevalence of DES or agree that students in the Middle East suffer from CVS more than their peers from other parts of the world, as investigators used ad hoc questionnaires rather than a validated one [1,2,15]. There are at least four questionnaires that either report symptoms of CVS or DES on a Likert scale or are Rasch-based, with few studies reporting on DES using one of these validated questionnaires. As far as the authors’ knowledge, this is the first study to use the validated CVS-Q to report prevalence of CVS among university students of different specialties.

The majority of the students in the current study used DD for more than six hours per day, which has been shown to be one of the risk factors of developing CVS [16-18], especially in contact lens users [11]. It is of interest to note that a higher percentage of students in this study use DDs for more than six hours compared to students in the region [2,12]. This could be due to lag of accommodation which has been linked to computer use [19,20], in addition to microfluctuations in accommodation observed during use of computer screens [21]. However, binocular vision functions were not measured in the study, which would limit the certainty of the role of binocular function in the study’s results. Many students complained of headache and blurred vision, and to a lesser extent eye pain and double vision. All these symptoms are linked to the demanding effect of DD use on accommodation and vergence [22]. Despite the extended duration of DD usage, the majority of students reported interruptions in using DD with many taking breaks (approximately 20 seconds duration every 20 minutes while looking at a distance of approximately 20 feet) while using their DDs, which is a habit practiced by students in Egypt and Saudi Arabia [2,12]. The American Optometric Association advocates taking a break after 20 minutes of DD use and looking at a distance of 20 feet for a period of 20 seconds as a simple and effective method of alleviating DES [23]. Nevertheless, it has been shown that eye care professionals may be aware of some of the symptoms of CVS but are not sure of the consequences or management strategies [24]. Eye care providers need to be aware of this simple advice of taking breaks during DD use that would reduce the effects of DES without compromising productivity of the students [25,26]. However, rest breaks in the workplace are of various types; the conventional two breaks of 15 minutes per working day has been used, while extended breaks amounting to an extra 20 minutes in addition to the “conventional’ breaks have been reported [25]. Thus, the duration and type of break as advocated by the American Optometric Association could be easily understood and ultimately used by both eye care professionals and the public.

It is interesting to note that more than one-third of the students reported that they felt their eyesight is worsening. It has been noted that despite the high prevalence of DES, it is not known if eye care providers, namely optometrists, are translating the effects of DD use on the eye or how they examine DES in the clinical setting [8]. Thus, it is imperative for eye care providers to be aware of the need to assess DES in the clinical setting and provide proper advice to their patients, which would contribute to the reduction of prevalence of CVS.

More than half of the students surveyed indicated that use of digital devices can affect their health and lifestyle. Around one-third of the students complained of pain in their neck and shoulders, in addition to pain in the joints of arm and wrist. Few students reported difficulty in writing with pens after extended use of smartphones, in addition to reduced ability to hold objects well. Pain in shoulders and neck is one of the most common symptoms reported by computer users [20]. Upper musculoskeletal pains are commonly experienced by DD users [26]. Despite the existing awareness of the effect of DD use on their health, fewer students showed their willingness to reduce digital device use. Increased awareness and reduced willingness to adjust current usage behavior are in agreement with previous reports on university students [2,27]. In contrast, other studies highlighted reduced awareness of CVS among university students [28]. Based on the aforementioned argument, it could be concluded that Jordanians, in general, are aware of the debilitating effects of certain conditions on their health; however, they are less inclined to comply with instruction or treatments [29]. Awareness of effects of digital devices among the public, particularly students, should be among the agenda of stakeholders and decision-makers. It is of interest that more females were more concerned about the effects of DD on their eyes and that they were more inclined to reduce their use of DD. A study related to contact lens prescribing in Jordan noted that the influence of culture in the Middle East would justify the difference between males and females in aspects related to ocular and general health [29].

## Conclusions

This study has established the prevalence of computer vision syndrome among the general university student population using a validated questionnaire, which supports the current evidence that university students are at a high risk of developing digital eye strain. Despite the fact that many students are aware of the effect of DD on their eyes and health, not as many are willing to adjust their usage behavior. One of the limitations of the study is that it encompassed students from one university which would not be representative of the student population in Jordan, and could perhaps over-estimated the prevalence of CVS among university students. Nevertheless, this is one of the few studies on prevalence of CVS that have used a validated CVS questionnaire. Data obtained from valid questionnaires are desirable as it allows decision-makers and stakeholders to plan their decisions and recommendations with confidence. It is recommended that well-informed awareness campaigns should be implemented to encourage healthier usage of DD to reduce the prevalence of DES and its associated ocular complications. As the world was affected by the COVID-19 epidemic, forcing the majority of education providers to move to online education, it is crucial to raise awareness of DES among students and educators.
